# Clinical Implications of Circulating Circular RNAs in Lung Cancer

**DOI:** 10.3390/biomedicines10040871

**Published:** 2022-04-08

**Authors:** Sae Seul Choi, Sae Eun Kim, Seon Young Oh, Young-Ho Ahn

**Affiliations:** 1 Department of Medicine, College of Medicine, Ewha Womans University, Seoul 07804, Korea; 1379013790@ewhain.net (S.S.C.); kse096@ewhain.net (S.E.K.); 2 Department of Molecular Medicine, Ewha Womans University, Seoul 07804, Korea; seonyoungoh@ewhain.net; 3 Inflammation-Cancer Microenvironment Research Center, College of Medicine, Ewha Womans University, Seoul 07804, Korea

**Keywords:** circular RNAs, lung cancer, liquid biopsy, diagnosis, prognosis

## Abstract

Circular RNAs (circRNAs) are single-stranded RNAs with a covalently closed-loop structure that increases their stability; thus, they are more advantageous to use as liquid biopsy markers than linear RNAs. circRNAs are thought to be generated by back-splicing of pre-mRNA transcripts, which can be facilitated by reverse complementary sequences in the flanking introns and trans-acting factors, such as splicing regulatory factors and RNA-binding factors. circRNAs function as miRNA sponges, interact with target proteins, regulate the stability and translatability of other mRNAs, regulate gene expression, and produce microproteins. circRNAs are also found in the body fluids of cancer patients, including plasma, saliva, urine, and cerebrospinal fluid, and these “circulating circRNAs” can be used as cancer biomarkers. In lung cancer, some circulating circRNAs have been reported to regulate cancer progression and drug resistance. Circulating circRNAs have significant diagnostic value and are associated with the prognosis of lung cancer patients. Owing to their functional versatility, heightened stability, and practical applicability, circulating circRNAs represent promising biomarkers for lung cancer diagnosis, prognosis, and treatment monitoring.

## 1. Introduction

Despite advancements in breakthrough therapies such as targeted therapy and immunotherapy, the survival rate of lung cancer patients has failed to improve for decades. Lung cancer remains the leading cause of cancer-related death worldwide, while the 5-year survival rate of patients with distant metastatic lung cancer is only 6% [1]. Thus, these outcomes urgently require the development of an effective early diagnosis method for lung cancer. Currently, early screening methods for lung cancer in clinical practice include sputum cytology, low-dose chest computed tomography, and autofluorescence bronchoscopy [2]. For the minimal or non-invasive early diagnosis of lung cancer, blood-based biomarkers are rapidly emerging as new alternatives and include circulating tumor cells, exosomes, and circulating nucleic acids (DNAs, microRNAs, and non-coding RNAs) [3,4].

Circular RNAs (circRNAs) are single-stranded endogenous RNAs with a covalently closed-loop structure [5]. Since their discovery in the 1970s, the study of circRNAs has been limited; however, with the development of next-generation sequencing, over the past decade, there has been an increased interest and research in circRNAs. As the physiological and pathological functions of circRNAs have been discovered, many studies have attempted to use circRNAs as biomarkers in the diagnosis of cancer. Owing to their stable structure [5], circulating circRNAs are thought to be more advantageous than normal linear RNAs for application in liquid biopsies. In this review, we briefly discuss the biogenesis and functions of circRNAs and describe in depth the recent findings and prospects for the application of circulating circRNAs in lung cancer diagnosis. More than 80 research and review articles were examined for the preparation of this manuscript. circRNA information including genomic positions and neighboring host genes was obtained from circRNA databases such as CircBank (http://www.circbank.cn; accessed on 28 February 2022) [6] and circBase (http://www.circbase.org; accessed on 28 February 2022) [7].

## 2. Discovery of circRNAs

Covalently closed circRNAs are not novel or unusual molecules in nature: in fact, the discovery of circRNAs was reported in the 1970s. Electron microscopy and biochemical analysis revealed that viroids, which are uncoated pathogenic RNA molecules that infect plants such as tomatoes, possess a circular structure [8]. The hepatitis delta virus likewise possesses a circular RNA [9]. Not limited to viruses, the linear intervening sequence of ribosomal RNAs in tetrahymena, a unicellular ciliate, could be converted to a circular shape upon heat shock [10]. Circularization of the *Sry* RNA transcripts has also been reported in murine testis, which might prevent SRY protein translation [11]. Circular *Fmn* RNA transcripts were identified in murine kidneys and contribute to the renal agenesis phenotype [12]. Similarly, some transcript variants of *ANRIL* non-coding RNA in humans were proven to be circular RNAs and were found to be associated with *INK4/ARF* expression and atherosclerotic vascular disease susceptibility [13]. Despite the widespread distribution of circRNAs from viruses to humans, RNA circularization has been considered an abnormal and unique phenomenon that only appears under special circumstances such as in the presence of unusual genomic structures surrounding host genes [11].

Through RNA sequencing and genomic annotation algorithms to extract “head-to-tail” spliced reads, thousands of circRNAs have been detected and identified in the human and murine transcriptomes over the last decade [14,15]. Indeed, an example is *CDR1as*, which functions as a microRNA (miRNA) sponge with its 74 seed matches against miR-7 [14,16]. Subsequent studies have revealed that circRNAs can function as regulators of alternative splicing and parental gene expression as well as miRNA sponges [17,18,19]. Through these diverse functions, circRNAs are involved in various physiological and pathological phenomena, which has made them the emerging focus of many research studies and fields, including cancer research.

## 3. Biogenesis of circRNAs

The formation of a circular structure via the covalent phosphodiester linkage between the 3′-hydroxyl group of a downstream exon and the 5′-phosphate group of an upstream exon is a seemingly sporadic event for RNA molecules. Although the exact mechanism through which circRNAs are generated has not been elucidated yet, circRNAs are considered to be generated with the help of specialized factors during the splicing process. An alternative “back-splicing” that joins together the 5′ GU donor site at the downstream intron and the 3′ AG acceptor site at the upstream exon promotes RNA circularization [20]. circRNAs can form from all precursor RNA regions including exons (protein-coding or untranslated regions) and introns.

During the typical mRNA splicing process, a lariat-structured intermediate molecule is produced [21], which can similarly generate circRNAs. The lariat formation catalyzes back-splicing by bringing the splicing sites closer together, thereby potentially providing an optimal situation for circRNA splicing [22]. However, circRNAs might also be produced through an alternative mechanism of direct back-splicing. Whereby, the back-splicing processes a pre-mRNA transcript into a circRNA along with an intermediate transcript composed of exons and introns, which is further processed into a linear RNA [23].

**Figure 1:**
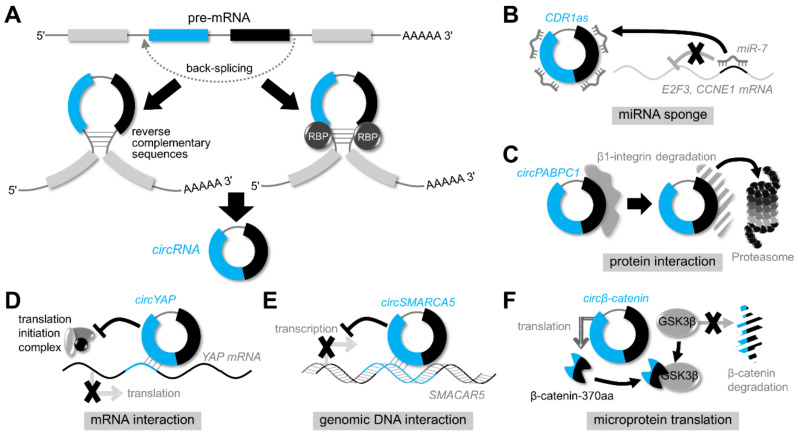
circRNA biogenesis and functions. (**A**) Biogenesis of circRNAs. circRNAs are produced through back-splicing from pre-mRNA transcripts. Back-splicing is facilitated either by reverse complement sequences in flanking introns or by RNA-binding proteins (RBPS) such as splicing regulatory factors, ESRP1, and QKI. (**B**–**F**) Functions of circRNAs. circRNAs function as miRNA sponges (**B**), interact with other proteins, RNAs, or genomic DNAs (**C**–**E**), or are translated into microproteins (**F**).

An alternative model for RNA circularization is through complementary sequence-mediated RNA paring across flanking introns (Figure 1A) [15]. Here, the production of normal linear mRNAs or circRNAs from a single precursor transcript could be determined by the competitive RNA paring between competitive sequences within individual introns or across flanking introns. All these models have been supported by evidence produced both in vitro and in vivo; however, it is still elusive how circRNAs are generated, although, importantly, circRNAs can be produced via different mechanisms depending on individual RNA transcripts or the surrounding molecular and cellular context [15].

circRNA biogenesis is precisely controlled by *cis*-regulatory elements and *trans*-acting factors, in addition to the spliceosome. Reverse complementary sequences in the flanking introns are particularly crucial in the production of circRNA [15,24]. The reverse complementary sequences are either repetitive sequences such as Alu elements, which are found in the flanking introns of 88% of circRNAs in humans [24], or non-repetitive sequences such as “GUUG” or “ACUU” regions participating in *circLONP2* production [25]. The pairing between reverse complementary sequences brings the downstream splice donor and upstream splice acceptor sites closer together, increasing the likelihood of back-splicing events.

RNA circularization can be facilitated by various *trans*-acting factors, which help bring the circle-forming exons together via the interaction with intronic sequence motifs. Spliceosome complexes (small nuclear ribonucleoproteins) and splicing regulatory factors (heterogeneous nuclear ribonucleoproteins and serine/arginine-rich proteins) regulate the production of circRNAs during the splicing of mRNA precursors [26]. Intronic reverse complementary sequences encourage the initiation of RNA circularization, while the splicing factors exquisitely control the expression levels of specific circRNAs. Indeed, the U2AF2 splicing factor binds to and facilitates the production of *circARF*, ultimately promoting glioma tumorigenesis [27]. Another splicing factor, ESRP1 expedited the production of *circUHRF1* through the interaction with intronic repeat sequences, which then promoted oral squamous cell carcinoma tumorigenesis [28]. In the mouse cortex, global circRNA biogenesis was suppressed by knocking out NOVA2, an RNA-binding protein regulating alternative splicing. NOVA2 was shown to bind to the flanking introns and facilitate back-splicing [29]. QKI is also known to bind to the flanking introns of circRNA-forming RNA precursors and promote circRNA formation during epithelial-to-mesenchymal transition in epithelial cancer cells [30]. Various *cis*-regulatory elements and *trans*-acting factors have been revealed to regulate the biogenesis of circRNAs [31]; however, the exact mechanism is still unclear, and more in-depth studies are required.

## 4. Functions of circRNAs

Like other regulatory non-coding RNAs, circRNAs interact with genomic DNA, mRNAs/miRNAs, and proteins to perform a variety of activities within cells (Figure 1B–F). As previously stated, the role of circRNAs as miRNA sponges has received the greatest attention. *CDR1as* is the first identified circRNA functioning as an efficient miRNA sponge that contains 74 miR-7-binding seed matches [14,16]. Upregulated in gastric cancer, *circNRIP1* was shown to work as a sponge against miR-149-5p and promote gastric cancer tumorigenesis and metastasis through the regulation of AKT/mTOR signaling [32]. In non-small cell lung cancer, *circPIP5K1A* (*circ_001430*) upregulates *BCL2* expression and thus inhibits apoptosis via sponging miR-136-3p [33].

circRNAs also bind to proteins and exert various functions. *circTNPO3* binds to and decoys IGFBP3 protein to prevent it from stabilizing *MYC* mRNA. *circTNPO3*, therefore, downregulates MYC and SNAIL expression and suppresses proliferation and metastasis in gastric cancer [34]. During neuronal differentiation, *circZNF827* was identified as a binding scaffold for a transcription-repressive complex composed of hnRNP-K/L proteins and the ZNF827 protein encoded from its host gene [35]. *circPABPC1* inhibited the migration and adhesion of hepatocellular carcinoma cells. Intriguingly, *circPABPC1* directly bound to and guided β1-integrin to the 26S proteasome, ultimately accelerating its degradation [36].

In addition, *circZNF609* directly interacts with several mRNAs including *CKAP5*, *UPF2*, and *SRRM1* mRNAs. *circZNF609* enhances the stability and translatability of these mRNAs by recruiting the ELAVL1 RNA-binding protein, which controls microtubule dynamics and drug resistance in rhabdomyosarcoma cells [37]. *circYAP* also specifically recognizes *YAP* mRNA and then suppresses its translation by inhibiting the assembly of the translation initiation machinery [38]. Furthermore, *circSMARCA5* directly binds to the genomic DNA of its host gene, *SMARCA5*, and suppresses its expression. The *SMARCA5* downregulation induced by *circSMARCA5* inhibited DNA damage repair and enhanced the drug sensitivity of breast cancer cells [39].

Interestingly, not all circRNAs are non-coding RNAs. In *Drosophila*, several circRNAs were found to be associated with ribosomes and translated into proteins [40]. *circSHPRH* (*circ_0001649*), which was downregulated in glioblastomas, suppressed tumorigenesis via its translational product, SHPRH-146aa [41]. In hepatocellular carcinoma, *circβ-catenin* promoted tumor growth and metastasis through the Wnt signaling pathway, which was mediated through translation into a novel isoform, β-catenin-370aa. This “microprotein” worked as a decoy for GSK3β, preventing the degradation of β-catenin [42]. Mechanistically, it was demonstrated that internal ribosome entry sites and 18S rRNA complementary sequences facilitate circRNA translation [43]. Some examples of circRNA translation that have been experimentally validated might be artifacts [44]; nevertheless, microproteins translated from specific circRNAs further expand the functional diversity and application range of circRNAs.

## 5. Circulating circRNAs

As previously described, circRNAs with diverse functions are involved in cancer development, progression, and metastasis. Like those of mRNAs, miRNAs, and proteins, the expression levels of circRNAs vary depending on the cell type; thus, they can be applied as diagnostic or prognostic markers in cancer patients. Numerous circRNAs have been reported to be upregulated or downregulated in various types of cancer. circRNAs have been discovered in plasma, saliva, urine, and cerebrospinal fluid, so that circulating circRNAs can be used as cancer biomarkers [45,46]. Like other linear RNAs, circRNAs can be amplified through reverse-transcription PCR (RT-PCR), which makes them more easily detectible than protein markers. Unlike linear RNAs, circRNAs lack free 5′- and 3′-ends, making them highly resistant to degradation by RNases with exonuclease activity.

It has been demonstrated that circRNAs are abundant and stable in exosomes, suggesting their significant translational potential as circulating biomarkers for cancer diagnosis [47]. In hepatocellular carcinoma, exosomal *circPTGR1* was shown to promote cancer progression through the regulation of the miR-449a/MET pathway [48]. Similarly, *circNRIP1* was also proven to be transmitted via exosomes and promoted tumorigenesis and metastasis of gastric cancer [32]. In laryngeal squamous cell carcinoma, *circRASSF2* was secreted by exosomes and promoted tumor growth through the regulation of the miR-302b-3p/IGF-1R pathway [49]. High *circCNOT2* expression was associated with poor progression-free survival of patients with breast cancer, and *circCNOT* is detectable in cell-free RNAs from patient plasma samples [50]. In addition, circRNAs can also be detected in circulating tumor cells [51]. Furthermore, circRNAs have been shown to be highly enriched in blood platelets compared with nucleated cells, which can be used for cancer diagnosis [52]. Since protein carriers such as high-density lipoprotein and Argonaute 2 transport miRNAs [53], the circulation of circRNAs might also be mediated by certain protein carriers or RNA-binding proteins.

Since there is a multitude of evidence indicating that circRNA could be useful in liquid biopsy, this review will highlight how circulating circRNAs can be used for the diagnosis and prognosis of lung cancer.

## 6. Functional Roles of Circulating circRNAs in Lung Cancer

### 6.1. Cancer Progression

The presence of *F-circEA* generated from the *EML–ALK* fusion gene was verified in non-small cell lung cancer (NSCLC) cells and in the plasma of NSCLC patients [54]. *F-circEA* promoted cancer cell migration and invasion, suggesting that *F-circEA* could be a novel liquid biopsy marker for NSCLC. Through circRNA profiling of serum or plasma obtained from patients, the clinical relevance of many circulating circRNAs has been explored (Table 1).

Global circRNA expression can be profiled by using RNA sequencing (RNA-seq) followed by bioinformatic approaches [72]. *circFARSA* was identified as an upregulated circRNA in NSCLC tissues compared with adjacent normal tissue by analyzing back-spliced reads on RNA-seq data [73]. *circFARSA* expression was higher in the plasma from NSCLC patients than in that from healthy volunteers and showed a good diagnostic value for NSCLC (AUC = 0.71). cDNA encoding *circFARSA* was cloned into the pLCDH-ciR vector, which was specifically designed to overexpress circular transcripts [55]. *circFARSA* overexpression enhanced the migration and invasion of A549 cells. Through in silico analyses, circFARSA was predicted to sponge miR-330 and miR-326 and regulate fatty acid synthesis. This is one of the earliest studies investigating the possibility of plasma circRNAs as new biomarkers for NSCLC patients; however, it lacks functional evidence supporting the molecular mechanism of *circFARSA* in NSCLC.

Through a microarray-based screening, *circYWHAZ* (*circ_0005962*) was identified as one of the upregulated circRNAs in lung adenocarcinoma (LUAD) [56]. The knockdown of *circYWHAZ* by siRNAs significantly suppressed the proliferation of LUAD cells, implying that this circRNA can promote cell proliferation [57]. Moreover, *circYWHAZ* expression was also upregulated in plasma samples, which illustrates a good diagnostic value for LUAD patients (AUC = 0.73). After surgical resection, *circYWHAZ* expression in the plasma decreased considerably, which suggests that *circ_0005962* is potentially a good noninvasive biomarker for LUAD diagnosis [57]. miRNA-target prediction and functional enrichment analysis showed that *circYWHAZ* might function as a miRNA sponge to regulate LUAD development, which needs further validation.

*circACP6* (*circ_0013958*) was also upregulated in LUAD tumors compared with nontumor tissues, which was validated by microarray and RT-PCR [58]. High expression of *circACP6* was associated with the TNM stage (*p* = 0.009, Cox analysis) and lymphatic metastasis (*p* = 0.006) in LUAD patients. Moreover, the plasma expression levels of *circACP6* distinguished LUAD from the control (AUC = 0.794, 95% CI = 0.703–0.912). Additionally, knockdown of *circACP6* inhibited the proliferation, migration, and invasion of LUAD cells. Mechanistically, *circACP6* functioned as a sponge against miR-134, which promoted the upregulation of cyclin D1, a target of miR-134. This study suggests that *circACP6* might also be a novel biomarker for LUAD [58]. In this study, the authors showed the functional mechanism and diagnostic value of plasma *circACP6*. However, since this study was conducted with a relatively small number of patients (*n* = 30), a follow-up study with a larger, more diversified group of patients is needed.

*circCXCR4* (*circ_0056616*) was identified and detected as a CXCR4-related circRNA in LUAD cells and exosomes [59]. Plasma exosome levels of *circCXCR4* were lower in LUAD patients with TNM stage III–IV or with lymphatic metastasis than in those with stage I–II or without metastasis, respectively. This suggests that *circCXCR4* might suppress the progression and metastasis of LUAD. Indeed, plasma exosomal *circCXCR4* represents a good biomarker to diagnose lymphatic metastasis of LUAD (AUC = 0.812, 95% CI = 0.720–0.903), which also needs to be validated in a larger group of patients.

Through the exoRBase database (http://www.exorbase.org; accessed on 3 June 2020), *circSATB2* (*circ_0008928*) was selected as a highly expressed circRNA in cancer exosomes [60]. The expression of *circSATB2* was higher in lung cancer cells than in normal bronchial epithelial cells. Furthermore, overexpression and knockdown experiments showed that *circSATB2* promoted the proliferation, migration, and invasion of lung cancer cells. Additionally, the packaging and transfer of *circSATB2* by exosomes influenced the proliferation and migration of the recipient cells. *circSATB2* directly bound to and inhibited miR-326, which in turn upregulated FSCN1, the presence of which has been reported as a poor prognostic marker for NSCLC patients [61]. Therefore, upregulation of FSCN1 by *circSATB2* via sponging miR-326 represents a potential mechanism through which *circSATB2* promotes NSCLC progression. In addition, serum exosomal *circSATB2* expression was higher in NSCLC patients with metastasis than in those without, demonstrating a good diagnostic value for metastatic NSCLC (AUC = 0.797, 95% CI = 0.698–0.896). This study clearly showed that *circSATB2* participated in NSCLC progression and could be a potential diagnostic marker for NSCLC.

In contrast, RNA-seq profiling demonstrated that *circ_0102537* was one of the downregulated exosomal circRNAs in LUAD, which was also retrieved from a microarray database (GSE101586). Moreover, *circ_0102537* was confirmed by quantitative RT-PCR to be downregulated in both plasma exosomes and tissues from LUAD patients. *circ_0102537* knockdown by siRNAs promoted the migration and invasion of lung cancer cells and enhanced the expression of EMT markers such as N-cadherin, Snail, and Vimentin. This suggests that *circ_0102537* might function as a tumor suppressor; however, the functional mechanism has not been presented [62]. Although many circulating circRNAs have been linked to lung cancer progression so far, further validation with more diverse groups of patients and in-depth mechanistic studies should be performed.

### 6.2. Anticancer Drug Response

Over a long period, numerous studies have been conducted to find predictive markers for sensitivity to EGFR inhibitors [74], and several circulating circRNAs have been proposed as candidate markers. Microarray analysis of plasma RNAs from NSCLC patients sensitive or resistant to gefitinib, an EGFR inhibitor, revealed that 1377 circRNAs were differentially expressed between the two groups [66]. Among them, *circZNF91* (*circ_0109320*) was upregulated in the gefitinib-sensitive group. The plasma levels of *circZNF91* could distinguish the gefitinib-sensitive group from the resistant group (AUC = 0.8054) and were associated with better progression-free survival in NSCLC patients treated with this EGFR inhibitor. Overall, *circZNF91* could be a predictive biomarker of the sensitivity to gefitinib treatment in NSCLC patients after comparative verification with other parameters in a wider and larger group of patients.

*circC3* (*circ_0002130*) increased in NSCLC cells that acquired resistance to the EGFR tyrosine kinase inhibitor, osimertinib [67]. *circC3* knockdown inhibited proliferation, glycolysis, and tumor growth in osimertinib-resistant lung cancer cells [68]. *circC3* acted as a sponge against miR-498 to upregulate its targets, GLUT1, HK2, and LDHA, which are glycolysis-related proteins. Furthermore, an increase in *circC3* was detected in serum exosomes from osimertinib-resistant NSCLC patients with respect to those from osimertinib-sensitive patients. *circC3* provided a good diagnostic value to predict the efficacy of osimertinib treatment in NSCLC patients (AUC = 0.792, 95% CI = 0.676–0.909), suggesting circulating *circC3* as a novel biomarker. A combination of two or more circulating circRNAs with other variables such as EGFR mutations and gene copy number [74] would be a better biomarker for predicting the sensitivity to EGFR inhibitors.

*circCNIH4* (*circ_0000190*) and *circSHPRH* were identified by RNA-seq to be upregulated in lung cancer cells compared with normal bronchial epithelial cells. They were also detected in conditioned media from lung cancer cells and in blood plasma samples by droplet digital PCR [63]. Furthermore, their plasma levels exhibited a poor response to immunotherapy, which might be due to the upregulation of soluble PD-L1 caused by these circRNAs [64]. Even though the detailed mechanism underlying the interplay between these circRNAs and antitumor immunity is still elusive, along with PD-L1 expression, their plasma levels could predict immunotherapy efficacy in lung cancer patients.

In-depth analysis of two GEO microarray datasets (GSE101684 and GSE101586) identified circRNAs highly expressed in LUAD samples compared with normal tissues [69]. Among them, *circ_002178* promoted PD-L1 expression via sponging miR-34a. *circ_002178* was also highly detected in plasma exosomes from LUAD patients compared with those from healthy volunteers, and exosomal *circ_002178* had a significant diagnostic value for LUAD (AUC = 0.9967). Intriguingly, *circ_002178* was transferred from cancer cells to CD8^+^ T cells via exosomes and then promoted PD-1 expression via sponging miR-28-5p. This indicates that *circ_002178* would be a good target for immunotherapy, since it can modulate the expression of PD-1/PD-L1 in LUAD. As shown in this section, circulating circRNAs are highly likely to be exploited as markers for predicting the responses to anticancer drugs once their mechanisms of action are confirmed and their efficacy is validated in more diverse patients.

### 6.3. Cancer Diagnosis and Prognosis

As noted previously, numerous circulating circRNAs (*circFARSA*, *circYWHAZ*, *circACP6*, *circSATB2*, *circZNF91*, *circC3*, and *circ_002178*) have significant diagnostic value and are associated with prognosis in lung cancer patients. In addition, RNA-seq and subsequent RT-PCR validation confirmed that *circCD226* (*circ_0047921*) and *circRALB* (*circ_0056285*) were downregulated, while *circATXN7* (*circ_0007761*) was upregulated in serum exosomes from NSCLC patients [70]. The combination of these three circRNAs provides a noteworthy diagnostic tool, which distinguishes NSCLC from healthy control (AUC = 0.919, 95% CI = 0.877–0.962) or other lung diseases, and their expression levels were associated with NSCLC progression. However, this study had several limitations such as insufficient sample sizes, samples from a single ethnic population, and lack of the mechanism of action of these exosomal circRNAs.

Plasma *circCNIH4* demonstrated diagnostic potentials in lung cancer patients at all TNM stages (AUC = 0.95 for stage I–IV, AUC = 0.896 for stage I–II, and AUC = 0.96 for stage III–IV) [63]. Patients with high plasma levels of *circCNIH4* exhibited poorer overall survival rates than those with low levels. Mechanistically, *circCNIH4* could modulate the EGFR/ERK pathway by sponging miR-142-5p [65]. *circPVT1* was also upregulated in tissues and sera from NSCLC patients. Both tissue and serum levels of *circPVT1* showed diagnostic potential, distinguishing NSCLC patients from controls (AUC = 0.803 and 0.794, respectively) [71]. The knockdown of *circPVT1* by siRNAs suppressed proliferation, migration, and invasion and promoted apoptosis in lung cancer cells. *circPVT1* facilitated E2F2 signaling by functioning as a sponge against miR-125b. Even though the authors did not present the effect of *circPVT1* on the survival or prognosis of NSCLC patients recruited in this study, they proved that *circPVT1* can be used as a diagnostic marker and elucidated its working mechanism in NSCLC.

The expression levels of several circulating circRNAs are associated with major mutations found in lung cancer. For example, *F-circEA*, but not its host linear mRNA, could be detected in EML4–ALK^+^ lung cancer plasma; thus, circulating *F-circEA* would be a novel biomarker to detect EML4–ALK fusion and to determine an effective treatment for EML4-ALK^+^ patients [54]. LUAD patients with high plasma expression of *circBNC2* (*circ_0086414*) were revealed to harbor EGFR mutations more frequently than those with low expression (*p* = 0.001) [57], suggesting that plasma *circBNC2* would be a companion diagnostic marker for EGFR tyrosine kinase inhibitors. Considering the examples described above and the stable structure of circRNAs, circulating circRNAs can be novel biomarkers for diagnosis, prognosis, and treatment monitoring in lung cancer patients.

## 7. Conclusions and Perspectives

Since circRNAs have only recently started to attract attention, much is still unknown about their biogenesis and mechanisms of action. Nevertheless, the functional diversity and broad application potential of circRNAs offer new opportunities in cancer diagnosis and prognosis. In particular, the circulating circRNAs presented in this paper have great potential versatility as candidates for non-invasive biomarkers. Unlike mRNAs and miRNAs [75], the heightened stability of circRNAs derived from their intrinsic structure further supports their applicability in liquid biopsy. However, there are several problems and limitations that must be addressed before their practical clinical application.

Basically, the expression levels of circulating circRNAs are quite low, so amplification and detection techniques with high sensitivity and accuracy are required. Techniques currently used for detecting circulating tumor DNA based on next-generation sequencing, digital-PCR, real-time PCR, or mass spectrometry could be applied to circulating circRNA detection [76]. Upregulation of plasma *circCNIH4* and *circSHPRH* in lung cancer patients was detected using droplet digital PCR [63] which can quantitate nucleic acids with high sensitivity and accuracy [77]. The NanoString nCounter^®^ platform, which is a molecular barcoding system with target-specific oligonucleotide probes, was also used for circRNA quantification [78]. The tethered cationic liposome nanoparticle biochip has shown high sensitivity and selectivity for exosomal miRNA detection in small volumes of patients’ serum [79], so it can also be applied for circRNA detection.

Despite the functional versatility and practical applicability of circulating circRNAs, there are still challenges to overcome before they can be applied in clinical settings, as has occurred for other liquid biopsy markers [75]. Since the exact mechanisms of action have not yet been elucidated, the expression levels of circRNAs that will actually lead to meaningful functional aspects are unknown. Depending on the purposes and scopes of application, it is necessary to determine whether qualitative or quantitative, or absolute or relative levels of circulating circRNAs are critical. The association of circRNAs with previously well-defined prognostic and diagnostic variables such as the Lung Cancer Prognostic Index (including stages, histology, mutation status, performance status, etc.) [80] should be considered in more depth. The relationship between circRNAs and key signaling players (e.g., KRAS, EGFR, BRAF, LKB1, MET, etc.) [81], which are closely related to lung cancer progression, should be studied in more detail.

Essentially, circulating circRNAs will face the same limitations and problems that previous candidate markers for liquid biopsy have encountered [82]. Firstly, since these cancer-associated markers are present in body fluids at very low levels, fast, cheap, and ultrasensitive detection techniques are required. The detection of circRNAs in exosomes or body fluids is also more time-consuming than assays based on conventional protein tests, which may limit the widespread use of circRNAs as biomarkers in clinical applications [51]. Proper samples should be accurately collected under tightly controlled environments with well-organized, systematic, efficient, and detailed protocols. Further, it should be clearly distinguished whether cancer cells or normal cells are the sources of the biomarkers. Some circRNAs can be expressed differently in tissues, although their serum levels are not significantly different. As in the case of alpha-fetoprotein in hepatocellular carcinoma, one of the most intensively studied cancer biomarkers [83], even though circRNA candidates are upregulated or downregulated in cancer tissues compared with non-cancer tissues, the difference between their plasma or serum levels in patients and healthy controls may not be apparent. These circRNAs are not suitable for diagnosing or predicting diseases in a non-invasive manner [51]. To be successfully integrated into clinical practice, the entire process from sample acquisition to marker analysis should be standardized and validated. 

As mentioned above, various circulating circRNAs control the progression of lung cancer by sponging cancer-related miRNAs or by affecting the expression of cancer-related genes. In order to apply circulating circRNAs to the treatment of lung cancer, additional research on the detailed mechanisms through which circRNAs inhibit cancer progression is necessary. Furthermore, even if treatment targets and the related pathways are identified, biological safety should be guaranteed for their practical application in cancer treatment. For example, nanoparticles are convenient for carrying circRNA plasmids or circRNA-targeting agents in animal models, but it has not been fully revealed how safe they are for clinical use [84]. In addition, synthetic circRNAs can boost the immune system in vivo because foreign circRNAs can be distinguished from endogenous circRNAs due to their lack of N6-methyladenosine modification [85]. There are still many obstacles in developing a treatment for lung cancer using circulating circRNAs; therefore, their application for diagnosis would be prioritized over their use in the treatment of lung cancer. Despite all these challenges, circulating circRNAs have advantages as promising biomarkers and can be actively used for lung cancer diagnosis, prognosis, and treatment monitoring.

## Figures and Tables

**Table 1:** List of circulating circRNAs in lung cancer.

circRNAs	Host Genes	Functions in Lung Cancer	Ref.
*F-circEA*	*EML4-ALK*	Promotes cell migration and invasion Associated with EML4–ALK fusion	[54]
*circFARSA*	*FARSA*	Upregulated in NSCLC tissues (a diagnostic marker) Promotes cell migration and invasion Sponges miR-330/miR-326 and regulates fatty acid synthesis	[55]
*circYWHAZ* *(circ_0005962)*	*YWHAZ*	Upregulated in LUAD, downregulated after surgical resection Promotes cell proliferation	[56,57]
*circACP6* *(circ_0013958)*	*ACP6*	Upregulated in LUAD (a diagnostic marker) Associated with TNM stages and lymphatic metastasis Promotes cell proliferation, migration, and invasion Sponges miR-134 and upregulates cyclin D1	[58]
*circCXCR4* *(circ_0056616)*	*CXCR4*	Downregulated in LUAD (stage III–IV or lymphatic metastasis) Suppresses LUAD progression and metastasis	[59]
*circSATB2* *(circ_0008928)*	*SATB2*	Upregulated in lung cancer cells (in exosomes) Promotes cell proliferation, migration, and invasion Sponges miR-326 and upregulates FSCN1 Increases in metastatic NSCLC (a diagnostic marker)	[60,61]
*circ_0102537*	*None (intergenic)*	Downregulated in LUAD (in exosomes) Suppresses EMT, cell migration, and invasion	[62]
*circCNIH4 (circ_0000190)*	*CNIH4*	Upregulated in lung cancer cells, upregulates soluble PD-L1 Associated with poor response to immunotherapy Associated with TNM stages and poor survival rates Sponges miR-142-5p and modulates EGFR/ERK signaling	[63,64,65]
*circSHPRH*	*SHPRH*	Upregulated in lung cancer cells, upregulates soluble PD-L1 Associated with poor response to immunotherapy	[63,64]
*circZNF91* *(circ_0109320)*	*ZNF91*	Upregulated in gefitinib-sensitive NSCLC Associated with better PFS in patients treated with gefitinib	[66]
*circC3* *(circ_0002130)*	*C3*	Upregulated in osimertinib-resistant NSCLC (in exosomes) Promotes cell proliferation, glycolysis, and tumor growth Sponges miR-498 and upregulates GLUT1, HK2, and LDHA Predicts the efficacy of osimertinib treatment	[67,68]
*circ_002178*		Upregulated in LUAD (in exosomes, a diagnostic marker) Sponges miR-34a and upregulates PD-L1 Transferred to CD8+ T cells and promotes PD-L1 expression via sponging miR-28-5p	[69]
*circCD226* *(circ_0047921)*	*CD226*	Downregulated in NSCLC (in exosomes) A diagnostic and prognostic marker	[70]
*circRALB* *(circ_0056285)*	*RALB*	Downregulated in NSCLC (in exosomes) A diagnostic and prognostic marker	[70]
*circATXN7* *(circ_0007761)*	*ATXN7*	Upregulated in NSCLC (in exosomes) A diagnostic and prognostic marker	[70]
*circPVT1*	*PVT1*	Upregulated in NSCLC (diagnostic marker) Promotes cell proliferation, migration, and invasion, suppresses apoptosis Sponges miR-125b and activates E2F2 signaling	[71]
*circBNC2* *(circ_0086414)*	*BNC2*	Associated with EGFR mutations A companion diagnostic marker for EGFR TKIs	[57]

## Data Availability

No new data were created or analyzed in this study. Data sharing is not applicable to this article.
